# A Novel Function of DELTA-NOTCH Signalling Mediates the Transition from Proliferation to Neurogenesis in Neural Progenitor Cells

**DOI:** 10.1371/journal.pone.0001169

**Published:** 2007-11-14

**Authors:** Barbara Hämmerle, Francisco J. Tejedor

**Affiliations:** Instituto de Neurociencias, Consejo Superior de Investigaciones Científicas (CSIC) and Universidad Miguel Hernandez, Sant Joan, Alicante, Spain; Ecole Normale Superieure, France

## Abstract

A complete account of the whole developmental process of neurogenesis involves understanding a number of complex underlying molecular processes. Among them, those that govern the crucial transition from proliferative (self-replicating) to neurogenic neural progenitor (NP) cells remain largely unknown. Due to its sequential rostro-caudal gradients of proliferation and neurogenesis, the prospective spinal cord of the chick embryo is a good experimental system to study this issue. We report that the NOTCH ligand DELTA-1 is expressed in scattered cycling NP cells in the prospective chick spinal cord preceding the onset of neurogenesis. These *Delta-1*-expressing progenitors are placed in between the proliferating caudal neural plate (stem zone) and the rostral neurogenic zone (NZ) where neurons are born. Thus, these *Delta-1*-expressing progenitors define a proliferation to neurogenesis transition zone (PNTZ). Gain and loss of function experiments carried by electroporation demonstrate that the expression of *Delta-1* in individual progenitors of the PNTZ is necessary and sufficient to induce neuronal generation. The activation of NOTCH signalling by DELTA-1 in the adjacent progenitors inhibits neurogenesis and is required to maintain proliferation. However, rather than inducing cell cycle exit and neuronal differentiation by a typical lateral inhibition mechanism as in the NZ, DELTA-1/NOTCH signalling functions in a distinct manner in the PNTZ. Thus, the inhibition of NOTCH signalling arrests proliferation but it is not sufficient to elicit neuronal differentiation. Moreover, after the expression of *Delta-1* PNTZ NP continue cycling and induce the expression of *Tis21*, a gene that is upregulated in neurogenic progenitors, before generating neurons. Together, these experiments unravel a novel function of DELTA–NOTCH signalling that regulates the transition from proliferation to neurogenesis in NP cells. We hypothesize that this novel function is evolutionary conserved.

## Introduction

One of the greatest challenges in the field of neural development is to elucidate how developmental signals are integrated to generate the wide cellular diversity of the brain. In order to generate the correct number of cells in the proper places, the balance between cell proliferation and differentiation must be regulated in a very precise spatio-temporal manner during brain development.

The vertebrate CNS originates from a relatively small number of founder progenitor cells. At early developmental stages, the number of progenitors expands in an exponential manner through a series of proliferative divisions. Subsequently, NP cells begin to generate neurons through neurogenic divisions that give rise to a new progenitor and a neuron, [1]–[6]. Thus, the whole developmental process of neurogenesis comprises several cellular steps including the switch to neurogenic NP cells, the cell cycle exit after division of at least one of the daughter cells, and its differentiation into a neuron or glial cell [7]–[10]. Therefore, a complete account of neurogenesis involves understanding a number of complex underlying molecular processes. Numerous molecular mechanisms involved in the regulation of the asymmetric division of NP [11], [12], cell cycle exit of neural cells [13], [14] and neuronal differentiation [15] have been extensively studied. On the other hand, the genes and molecular processes that govern the switch from proliferative to neurogenic NP cells remain mostly unknown and, furthermore, little is known of how these sequential steps are coordinated.

Signalling through the NOTCH receptor is essential for correct cell-fate specification and differentiation throughout the animal kingdom [reviewed in 16], [17]. The NOTCH proteins are cell-surface transmembrane receptors that upon binding to their ligands (DELTA, SERRATE, and JAGGED) located on the surface of adjacent cells, transduce a signal that influences cell fate choices. Several lines of evidence show that NOTCH signalling is involved in regulating neurogenesis in the vertebrate nervous system [reviewed in 18], [19]. This takes place by the mechanism known as NOTCH mediated lateral inhibition. In brief, *Delta-1* is expressed in single cells, which differentiate into neurons, and it impairs the differentiation of neighbouring cells by activating NOTCH signalling. Following DELTA binding to the NOTCH receptor in the neighbouring cells, NOTCH is cleaved and its intracellular domain (NICD) translocates to the nucleus, where it stimulates the expression of the *Hes* family of bHLH transcription factors. In turn, these factors repress the expression of proneural bHLH transcription factors leading to the inhibition of neuronal differentiation. At the same time, the expression of *Delta* is inhibited in these cells and this decreases NOTCH activity in the *Delta* expressing cell by the feedback loop mechanism of lateral inhibition. Thus, the inhibited cells remain as progenitors and the *Delta* expressing cell differentiate as a neuron. This is the mechanism that was originally proposed to happen in the rostral NZ of the developing chick spinal cord [20]. Nevertheless, DELTA-NOTCH signalling fulfils a different role in the growing caudal neural plate of the chick embryo. In this region, both *Notch* and *Delta-1* are widely expressed in uncommitted progenitor cells. This leads to mutual NOTCH signalling which serves to maintain the proliferating progenitor pool necessary for the caudal extension of the body axis [21], [22].

Here, we present compelling evidence for a new function of DELTA-NOTCH signalling, which regulates the transition from proliferation to neurogenesis in the prospective spinal cord of the chick embryo. This signalling takes place among NP cells which are located in between the caudal neural plate (stem zone) and the rostral NZ as the body axis extend caudally.

## Results

### In the developing spinal cord, *Delta1* is expressed in cycling neural progenitor cells preceding the onset of neurogenesis

The prospective spinal cord of the chick embryo presents a well defined rostro-caudal gradient of neurogenesis [23]–[25]. This yields a sequential separation of the cellular processes of proliferation and neurogenesis along the rostro-caudal axis [reviewed in 26]. Thus, this is a particularly suitable experimental system to investigate the molecular mechanisms underlying the transition from proliferation to neurogenesis. Although there are some small populations of neurons that appear very early in development [27], the main onset of neurogenesis in the chick embryo takes place after the closure of the neural tube [24]–[25].

**Figure 1:**
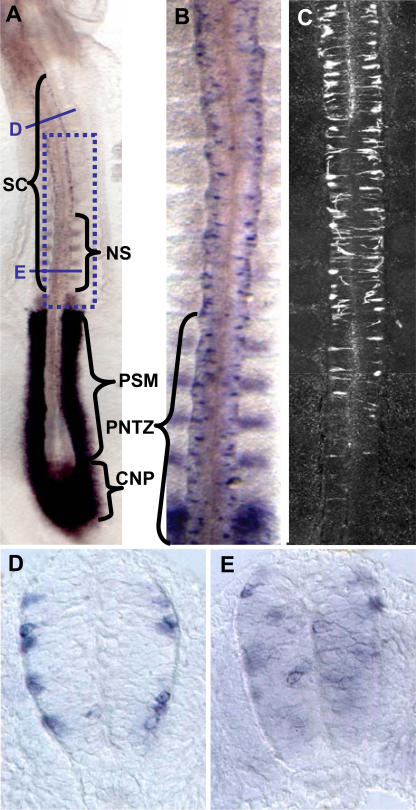
Differential rostro-caudal pattern of *Delta-1* expression in the prospective chick spinal cord and its spatial correlation to neurogenesis. A. ISH of Delta-1 in a HH11 chick embryo showing expression in the caudal neural plate (CNP), the last formed caudal somites (NS), the prospective spinal cord (SC), and the presomitic mesoderm (PSM). B. Higher magnification of the framed area in A showing Delta-1 expression in scattered cells along the prospective spinal cord. C. Immunofluorescent TUJ1 labelling of a HH11 chick embryo in a region equivalent to panel B. Note that very few labelled cells are detected in the region of the prospective spinal cord around the last formed caudal somites (PNTZ, proliferation to neurogenesis transition zone). In contrast there is a large number of *Delta-1*-expressing cell in the equivalent region of panel B. D,E. Transverse sections at approximately the positions indicated in panel A. Note the different apico-basal positions of labelled cells between the rostral and caudal sections.

**Figure 2:**
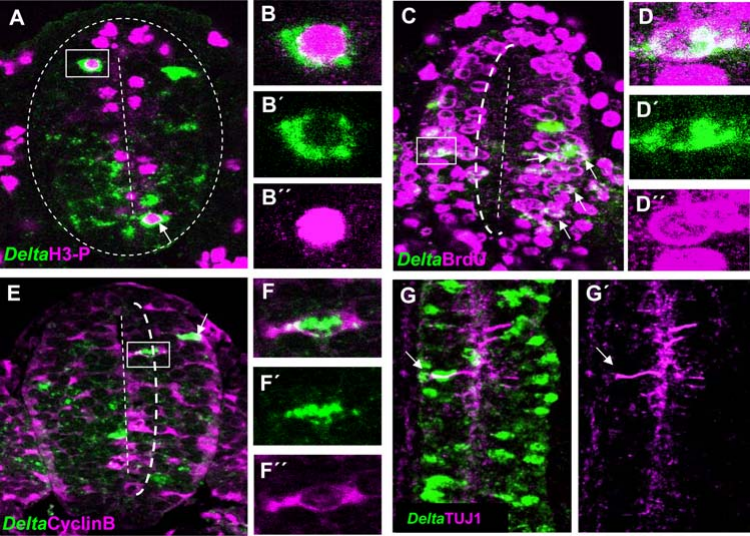
*Delta-1* is expressed in cycling neural progenitor cells. Double immunolabelling of *Delta-1* mRNA and cell cycle markers in transverse sections of the prospective spinal cord at the level of the last five caudal somites in HH10 chick embryos. Confocal optical sections of 6 µm are shown and double labelled cells are indicated by arrows. A. Several cells co-expressing *Delta-1* mRNA and PH3 are located near the ventricle lumen B–B″. Higher magnification of the boxed cell in A. C. A large proportion of *Delta-1* expressing cells incorporate BrdU after a single pulse. Also note that practically no BrdU labelled cells are located within 1/3rd of the apical-basal distance from the ventricle (dotted line). An example of a *Delta-1*/BrdU-double labelled cell is shown at higher magnification in D–D″. E. Cells co-expressing Cyclin B and *Delta-1* can be found within 1/3 of the apical-basal distance from the ventricle (dotted line). F–F″. Higher magnification of the boxed cell in E. G,G′. Confocal projection (dorsal view) over 20 µm taken from the prospective spinal cord of an HH10 embryo at the level of somites 7–8^th^ showing expression of Delta-1 and TUJ1. Note that very few double labelled cells (arrows) can be seen.

The neurogenic gene *Delta-1* is expressed in an interesting rostro-caudal pattern along the prospective spinal cord of the chick embryo (Fig. 1A,B). In agreement to its role in neuronal differentiation [16], [19], [20], abundant *Delta-1*-expressing cells are present in the rostral NZ of the prospective spinal cord where neurons are born according to the expression of the early neuronal marker, class III β-tubulin (TUJ1) [28]. Intriguingly, we found numerous scattered *Delta-1*-expressing cells in the caudal region where neurons are practically absent (compare Fig. 1B and C). Therefore, it appears that *Delta-1* expression in single cells of the caudal spinal cord precedes the onset of neurogenesis. Thus, we set out to determine the nature of these *Delta*-expressing cells. Interestingly, we observed that cells expressing *Delta-l* were found at different apico-basal positions along the rostro-caudal axis. In the NZ (i.e. between somites 1–4 in a HH11 embryo), they were located preferentially in basal positions (Fig. 1D), in accordance with its expression in prospective neurons [20] that move to the mantle as they withdraw from the cell cycle. On the other hand, in the caudal region, they were found in all apico-basal positions of the neuroepithelium (Fig. 1E), suggesting that *Delta-1* may be expressed by NP cells of the caudal spinal cord. Cycling NP cells are subject to interkinetic movements and hence, their apico-basal positions depend on the cell cycle phase [29]. To confirm this hypothesis, the coexpression of *Delta1* with distinct cell cycle markers was analysed in the caudal spinal cord. The expression of *Delta1* was assayed in mitosis (M phase), S phase, and interphase G2 using antibodies against phosphohistone-3 (PH3), BrdU, and cyclin B, respectively. As a result, we found that *Delta-l* is expressed in the M, S, and G2 phases of the cell cycle in spinal cord NP cells (Fig. 2 and Table 1). Since there is no suitable antiserum available for immunocytochemistry against chick cyclin D that allowed to test the expression in interphase G1, the coexpression of cyclin D and *Delta1* in NP cells was assayed in preneurogenic regions of the developing mouse brain where *Delta-1* expression has been also observed in scattered cells [30]. We indeed found a consistent proportion (32±5%, 3 embryos; 47/146 cells) of *Delta-1*/CYCLIN D double labelled cells in the forebrain of E10.5 mouse embryos. (Figure S1). Thus, it seems likely that *Delta-1* is also expressed during the G1 phase in spinal cord NP cells. As expected, there were relatively very few *Delta-1*/TUJ1 double labelled cells in this region (Table 1 and Fig. 2G).

**Table 1:** Quantitative analysis of *Delta-1* expression with cell cycle markers.

Cell cycle phase	Marker	n	N_m_/N_Dl1_	% (N_m_/N_Dl1_)±SD
M	Phosphohistone-3	3	31/241	13±1
S	BrdU	3	54/232	23±4
G2*	Cyclin B	3	37/188	19±3
postmitotic	TUJ1	3	10/202	5±2

Counting of double labelled cells was carried out on confocal images collected from transverse sections of HH10 chick embryos at the level of the last five caudal somites.

* Counting of *Delta-1*/Cyclin B double labelled cells was restricted to the apical third of the neuroepithelium that does not incorporate BrdU (see dotted line in Fig. 2C,E).

n: number of embryos analysed. N_Dl1_: number of *Delta-1*-expressing cells. N_m_ number of cells co-expressing *Delta-1* and the indicated marker. The error was calculated as the standard deviation (SD).

Together, these results show that the caudal spinal cord *Delta-1*-expressing cells are mostly cycling NP cells that define an intermediate domain between the rostral domain of *Delta-1* expression in prospective neurons (approximately rostral to the 5 last formed caudal somites) and that of the proliferating uncommitted progenitor cells of the caudal neural plate (Fig. 1A–C). Accordingly, we will call this domain the “proliferation to neurogenesis transition zone” or just PNTZ

### DELTA/NOTCH signalling in neural progenitor cells of the prospective spinal cord of early chicken embryos

**Figure 3:**
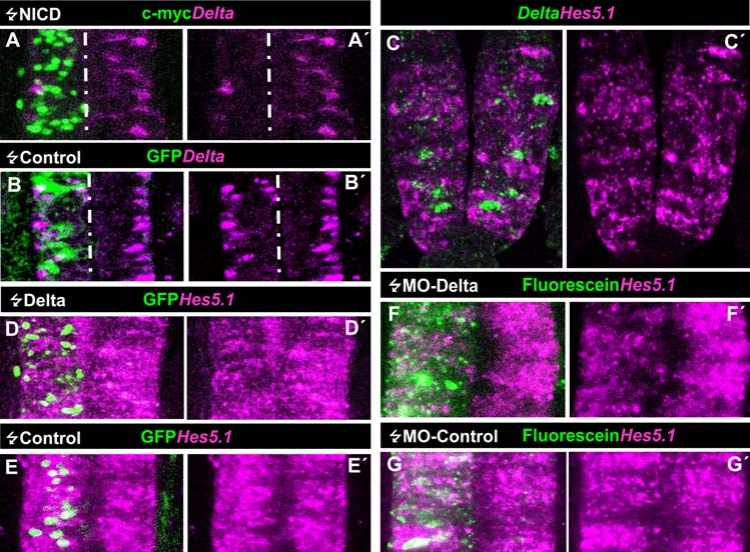
DELTA-1/NOTCH signaling in the caudal spinal cord of chick embryos. A–A′. Confocal projection over 50 µm from a dorsal aspect of the prospective spinal cord at the level of the 12^th^ and 13^th^ somites counted from rostral of a HH13 chick embryo that was electroporated with NICD at stage HH10 when that region was part of the PNTZ. Transfected cells express the c-myc reporter. Note the clear decrease in the number of *Delta-1* expressing cells within the electroporated area compared to the contralateral side and to the control transfected embryo (B–B′). C–C′. Transversal optical section of a double FISH of *Delta1* and *Hes5.1* taken at the level of the 12^th^ and 13^th^ somite. Notice that *Delta-1* and *Hes5.1*-expressing cells are mutually exclusive and that a few cells lack expression of both genes. D–D′. Confocal projection over 50 µm taken from a dorsal view point of the prospective spinal cord at the level of the 12th–14^th^ somites counted from rostral of an embryo that was electroporated with pCIG-*Delta1* at HH10 stage and allowed to develop until HH12/13 stage. Transfected cells express the reporter GFP protein. Notice the clear decrease in *Hes5.1* expression in *Delta-1* transfected cells while *Hes5.1* expression is maintained in most non transfected cells. In contrast, control pCIG transfected cells do not altere *Hes5.1* expression (E–E′). F–F′. Confocal projection over 50 µm taken from a dorsal view point of the prospective spinal cord at the level of the 12th–14^th^ somites counted from rostral of an embryo which was electroporated with the antisense morpholino oligo Mo2-cDelta1 at HH10 stage and allowed to develop until HH12/13. Notice the decrease in the expression level of Hes5.1 in the transfected side. as compared to the non-transfected contralateral side. In contrast, the pattern of *Hes5.1* expression is not altered in an embryo transfected with the control morpholino(G–G′).

The scattered expression pattern of *Delta-1* in the PNTZ suggests that these NP cells may be subject to DELTA-NOTCH lateral inhibition. In order to assess this idea, we tested whether the expression of *Delta-1* was under the control of NOTCH signalling. At this end, we transfected a constitutively active truncated form of NOTCH (NICD) [31], along with a pEGFP reporter plasmid, into the PNTZ of HH10 embryos. As shown in Fig. 3A,B, this resulted in an almost complete suppression of *Delta-1* expression (4/4 embryos).

In addition, we studied the relation of *Delta-1* with the expression of *Hes* genes, the primary transducers of NOTCH signals in vertebrates [reviewed in 32]. Among them, *Hes5.1* seemed to be the best candidate since it is abundantly expressed along the prospective spinal cord in a rostro-caudal distribution apparently overlapping with that of *Delta-1* (compare Fig. 1A and Figure S2). Double FISH shows that *Hes5.1* and *Delta-1* are expressed in a mutually excluding cellular pattern with low *Hes5.1* expression in *Delta1*-expressing cells and, conversely, high *Hes5.1* expression in the adjacent ones. (Fig. 3C). Nevertheless, we observed that a few cells expressed neither *Hes5.1* nor *Delta-1*. As shown in Fig. 3D,E, electroporation of the caudal spinal cord of HH10 chick embryos with *pCIG-Delta1* in scattered cells reproduced the endogenous expression pattern of *Hes5.1*. These results strongly suggest that DELTA1-NOTCH lateral inhibition takes place through *Hes5.1* in NP cells of the PNTZ

### Activation of NOTCH signalling in caudal spinal cord progenitor cells represses neurogenesis

**Figure 4:**
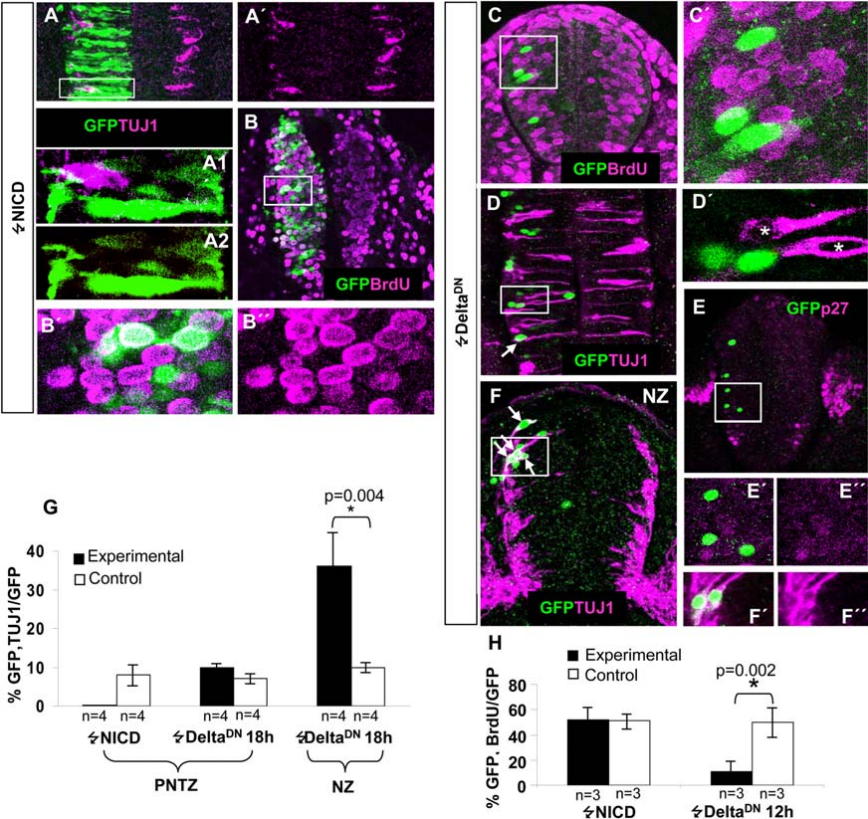
Effects of the activation and inhibition of NOTCH signalling on spinal cord progenitor cell proliferation and neuronal generation. A–A′. Confocal projection over 50 µm at the level of the 12^th^ and 13^th^ somites of an HH13 embryo that was electroporated with NICD at stage HH10. Note the clear decrease in the number of TUJ1-labelled cells within the electroporated area when compared to the contralateral side. A1–2. A high magnification of a single confocal section of the cell framed in A shows that in fact is not double labelled. B. Single 5 µm transversal confocal section of an embryo that was pulsed with BrdU 16 h after electroporation with NICD. Notice the large proportion of transfected cells that incorporated BrdU. B′,B″. High magnification of the framed area in B showing several double labelled cells. C. Partial confocal transverse projection (10 µm) of an embryo pulsed with BrdU 11 h after being electroporated with pCIG-*Delta^DN^*. C′. High magnification of the framed area in B showing the lack of GFP/BrdU positive cells. D. Confocal projection (50 µm) from a dorsal aspect of an embryo electroporated with pCIG-*Delta^DN^* at the HH10 stage and allowed to develop to the HH12/13 stage. Note the low incidence of GFP/TUJ1 positive cells (arrows). D′. Higher magnification of a single confocal optical section within the framed area in D showing two TUJ1+cells that lack GFP in their nuclei (asterisks) and two GFP labelled nuclei lacking TUJ1 in their soma. E. Partial confocal transverse projection (20 µm) of an embryo electroporated at the PNTZ with pCIG-*DeltaDN* allowed to develop further 18 h. E′ and E″ higher magnification of the boxed region showing that transfected cells lack p27KIP1 immunostaining. F. Confocal transverse projection (50 µm) of an embryo electroporated at the NZ with pCIG-*Delta^DN^* and allowed to develop further 18 h. F′–F″. Higher magnification of a single optical section (5 µm) of the boxed region of F showing two double labelled cells. G and H. Statistical analysis of embryos transfected with NICD, *Delta^DN^*, and control vectors.

Since NOTCH lateral inhibition has been shown to repress neuronal differentiation in several vertebrate nervous systems [reviewed in 19], [33], we determined whether the activation of NOTCH signalling causes similar effects in the caudal spinal cord. As a read out of neuron production we used the pan-neuronal marker TUJ1, which we have previously shown to be expressed as early as 1–2 h after mitosis of early chicken spinal cord NP cells [34]. NICD was transfected into the PNTZ of HH10 embryos around prospective 12^th^–16th somite pairs where neurogenesis had not commenced yet. We found that 20 hours after transfection (16 h after the initial detection of GFP, n = 4), NICD completely impaired the co–expression of GFP and TUJ1 in that region (0/216 cells: Fig. 4A–A2,G). These results indicate that NOTCH activation in PNTZ NP cells inhibit neurogenesis. In parallel experiments, transfected embryos were exposed to BrdU during the final 4 hours of incubation (n = 3, Fig. 4B). As shown in Fig. 4H, a similar proportion of GFP expressing cells incorporated BrdU in both NICD and control transfected embryos (320/617 and 58/109 cells, respectively).

### Inhibition of NOTCH signalling in caudal spinal cord neural progenitor cells arrests proliferation but does not elicit neuronal differentiation

The above results show that activation of NOTCH signalling in PNTZ NP cells represses neuronal production. Nevertheless, this does not seem to further stimulate the proliferation of these progenitors. Although this might be due to the fact that the expected increase (around a 10% of TUJ1 positive cells in the control, Fig. 4G) is in the range of the experimental error of the determination of BrdU labelled cells, these results raise the question of whether NOTCH signalling is required to maintain proliferation of these cells. In order to assess this idea, we analysed the effects of inhibiting NOTCH signalling in these cells by using a truncated form of DELTA with a deletion of the intracellular domain, which acts in a dominant negative manner [35]. This inhibition takes place by forming intracellular heteromeric complexes which interfere with NOTCH signal receptivity [36]. For instance, *Delta^DN^* has been previously shown to be an effective inhibitor of NOTCH signalling in chick nervous system [37], [38]. Thus, we transfected *Delta^DN^* into the PNTZ of HH10 embryos by electroporation and assayed proliferation by BrdU incorporation. In order to make sure of the cell autonomous effect of *Delta^DN^* we transfect few scattered cells. As expected, *Delta^DN^* induced a strong arrest of proliferation 12 h posttransfection (Fig. 4C–C′). Indeed, only about 10% of the *Delta^DN^* expressing cells incorporated BrdU (16/141) in comparison with the 48% of transfected cells that incorporated BrdU in control embryos (292/608: Fig. 4H). Thus, we conclude that NOTCH signalling is required to maintain proliferation of caudal spinal cord NP cells. Interestingly, the arrest in proliferation induced by *Delta^DN^* was not accompanied by a significant increase in the proportion of cells co-expressing TUJ1 18 h (4 embryos, 20/194 cells; 10,3%; Fig. 4D,D′,G) and 26 h (3 embryos, 14/149 cells; 9.4%, not shown): after transfection as compared to controls (4 embryos, 8/113 cells, 7.1%). The fact that a large part of the transfected cells were located on basal positions (Fig. 4D) may suggest that they were driven to exit the cell cycle by the expression of *Delta^DN^*. This possibility was assessed by analysing the expression of the cyclin-dependent kinase inhibitor *p27^kip1^*, which is a major regulator of cell cycle exit [14] and whose expression has been associated to the birth of neurons in the mouse forebrain [39] and the chick spinal cord [40]. We found that most *Delta^DN^* transfected cells lacked or exhibited very low *p27^kip1^* expression (Fig. 4E). This makes very unlikely that NP cells were removed from the cell cycle. An alternative explanation for the basal localization of the cells is that they were arrested in G1 by the decrease of NOTCH signalling as it happens when NOTCH signalling is inhibited by γ–secretase inhibitors [41]. The lack of suitable cyclin D antisera precluded to assess this possibility. Together, these experiments indicate that suppression of NOTCH signalling in caudal spinal cord NP cells arrests proliferation but it is not sufficient to elicit cell cycle exit and neuronal differentiation. This is in clear contrast to the induction of *p27^kip1^* (not shown) and TUJ-1 expression (4 embryos, 37,5% of double labelled cells vs. 9,8% in 4 control embryos; Fig. 4F,H) by *Delta^DN^* in the NZ (around prospective 3rd–7th somite pairs of HH12 embryos) 18 h after transfection.

### *Delta-1* expression in cycling neural progenitor cells is necessary and sufficient to induce neuronal generation

**Figure 5:**
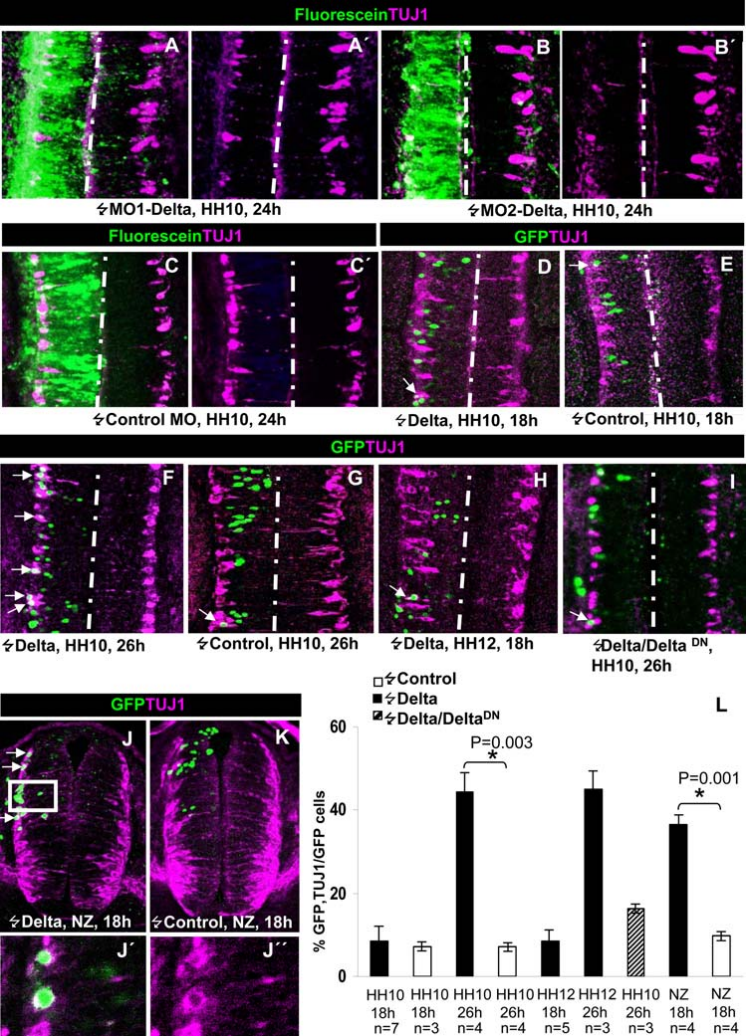
Spatio-temporal analysis of neuronal generation induced by *Delta-1* expression. A–I Confocal projections (40–50 µm) from a dorsal point of view of the prospective spinal cord of embryos transfected with pCIG-*Delta-1*, pCIG-*Delta-1*/pCIG-*Delta^DN^*, pCIG (controls), *Delta-1* anti-sense (MO1-c*Delta1* and MO2-c*Delta1*) and control morpholinos at the stages and times indicated. Arrows point to double labelled cells. J and K. Confocal transverse projections (50 µm) of two embryos electroporated at the NZ with pCIG-*Delta-1* and pCIG, respectively, and allowed to develop for 18 h. J′ and J″, higher magnification of a single optical section (5 µm) of the boxed area in J showing two GFP/TUJ1 labelled cells. L. Statistical analysis of the GFP/TUJ1 double labelled cells at the indicated stages and times after transfection with pCIG-*Delta-1,* pCIG*-Delta-1*/pCIG-*Delta^DN^* and pCIG (controls). Notice that a consistent increment in the proportion of double labelled cells are only obtained at 26 h postransfection with pCIG-*Delta-1* in PNTZ independently of the transfected embryonic stage

So far, we have shown that DELTA -NOTCH signalling in cycling NP cells precedes the onset of neuronal generation at the caudal spinal cord. Although suppression of NOTCH signalling induced proliferation arrest of these cells, it was not sufficient to elicit neuronal differentiation. Thus, it remains unclear how the expression of *Delta-1* in cycling NP cells is related to the process of neurogenesis and in what context of the different cellular steps along the rostro-caudal axis may be acting. To address these questions, we performed gain and loss of function experiments by focal electroporation at different rostro-caudal positions of the prospective spinal cord of stage HH10-HH12 chicken embryos. Experiments are summarised in Table 2. Loss of function was carried out by posttranscriptional gene silencing of *Delta-1* with two anti-sense and one control morpholino oligos (see Material and Methods for details). Since we have no available anti-DELTA-1 antisera to test the decrease of protein expression on the tissue, the efficiency of the *Delta-1* antisense morpholino oligos was assessed by analysing their effect on the expression of *Hes5.1*. As exemplified in Fig. 3F,G, the expression level of *Hes5.1* in the PNTZ was substantially reduced by electroporation of anti-sense morpholinos (4/5 embryos) while the control morpholino oligo did not modify the *Hes5.1* expression pattern (3/3 embryos). Accordingly, we tested the effect on neuronal generation. As shown in Fig. 5A–C, electroporation of morpholino anti-sense oligos induced an extensive decrease of TUJ1 immunolabelling whereas the control morpholino did not.

**Table 2:** Phenotypic analysis in gain and loss of function experiments of *Delta-1*.

Experiment	Region	Phenotype or Test	Exp. N_Ph_/N_T_	Control N_Ph_/N_T_	Figure
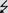 MO-Delta HH10+24 h	PNTZ	Decreased TUJ1 labelling	9/9	0/3	5A,B,C
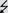 Delta HH10+18 h	PNTZ	Normal TUJ1 labelling	13/14	4/4	5D,E,L
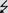 Delta HH10+26 h	PNTZ	Increased TUJ1 labelling	9/9	0/4	5FG,L
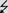 Delta HH12+18 h	PNTZ	Normal TUJ1 labelling	7/8	4/4	5H,L
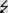 Delta HH12+26 h	PNTZ	Increased TUJ1 labelling	3/3	0/4	5L
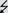 Delta HH10+18 h	PNTZ	Normal BrdU incorporation	6/6	4/4	6A,B,E
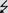 Delta HH10+26 h	PNTZ	Decreased BrdU incorp.	5/5	0/6	6C,D,E
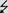 Delta HH10+18 h	PNTZ	Co-expression of PH3	3/3	3/3	6F
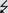 Delta HH10+18 h	PNTZ	Co-expression of Cyclin B	3/3	3/3	6G
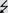 Delta^DN^ HH10+18 h	PNTZ	Normal TUJ1 labelling	4/4	4/4	4DG
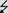 Delta/Delta^DN^ HH10+18 h	PNTZ	Normal TUJ1 labelling	3/3	3/3	N.S
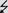 Delta/Delta^DN^ HH10+26 h	PNTZ	Normal TUJ1 labelling	3/3	3/3	5I
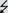 Delta/Delta^DN^ HH10+18 h	PNTZ	Decreased BrdU incorporation	3/3	3/3	N.S
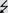 Delta/Delta^DN^ HH10+26 h	PNTZ	Decreased BrdU incorporation	3/3	3/3	6E
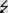 Delta^DN^ HH10+18 h	PNTZ	Normal p27KIP1 labelling	3/3	3/3	4E
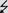 Delta^DN^ HH12+18 h	NZ	Increased p27KIP1 labelling	3/3	3/3	N.S.
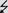 Delta HH12+18 h	NZ	Increased TUJ1 labelling	4/4	4/4	5 J
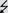 Delta HH12+26 h	NZ	Increased TUJ1 labelling	3/3	3/3	N.S.
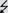 Delta^DN^ HH12+26 h	NZ	Increased TUJ1 labelling	3/3	3/3	4FG

The phenotypic analysis was carried out by Confocal Microscopy of whole mount embryos. N_Ph_: Number of embryos with clear phenotype. N_T_: Total number of analysed embryos.

N.S. = not shown.

In order to test the effects of *Delta-1* gain of function, we electroporated the pCIG-*Delta1* vector in the PNTZ. Nevertheless, as previously found in other chick neural tissues [37], [38], we observed that widespread transfection of cells with *Delta-1* inhibited neurogenesis in the PNTZ (Figure S3). This predictable inhibitory effect is explained by the mutual lateral induction of NOTCH signalling when many neighbour cells express both DELTA and NOTCH. To overcome this problem we used electroporation conditions for transfecting scattered cells with high levels of *Delta-1* expression, emulating the endogenous pattern of expression. Embryos were incubated for 10, 18 and 26 h, and TUJ1 labelling was analyzed in the transfected cells (Fig. 5D–G,L). The percentage of *Delta-1* transfected cells expressing TUJ1 10 h after transfection was less than 2% (not shown). 8 hrs later, there was no significant increase in the percentage of *Delta-1* transfected TUJ1 labelled cells (8.5±4% vs. 7±2%,). However, this effect increased greatly at 26 h after transfection (45±7%, vs. 7±1% in control embryos). Together with the electroporation of antisense morpholinos, these experiments demonstrate that the expression of *Delta-1* in NP cells of the PNTZ is necessary and sufficient to induce the generation of neurons. Interestingly, the onset of neuronal generation after *Delta-1* expression needs a longer period in the PNTZ than in the NZ where we measured a consistent increase in TUJ1 labelled cells 18 h after transfection of *Delta-1* (35,2±3,5%, vs. 9,8±0,6% in control embryos; Fig. 5J,K,L). Thus, neurons need approximately 8 and 16 h to arise in the NZ and PNTZ, respectively, after *Delta-1* expression if we consider that there was a good correlation between GFP and *Delta-1* mRNA expression at 8 h post-transfection (Figure S3), and TUJ1 can be detected as early as 1–2 h after mitosis [34]. These additional 8 h between the NZ and the PNTZ could reflect the time required by a rostro-caudal wave of differentiation to reach the transfected cells of the PNTZ. To assess this possibility, we transfected the same region (prospective somites 12–16) of older embryos (HH12) and allowed them to develop for 18 h, the time required to reach the same developmental stage (HH16) as HH10 embryos incubated for 26 hours. However, no increase in the percentage of TUJ1-positive cells was observed under these experimental conditions (Fig. 5H,L). On the other hand, a similar percentage of TUJ1-positive cells was obtained when both HH10 and HH12 *Delta-1* transfected embryos were allowed to develop for 26 h (Fig. 5L). These results rule out the possibility that a rostro-caudal gradient of differentiation might explain the delayed generation of neurons after *Delta-1* expression in the PNTZ.

**Figure 6:**
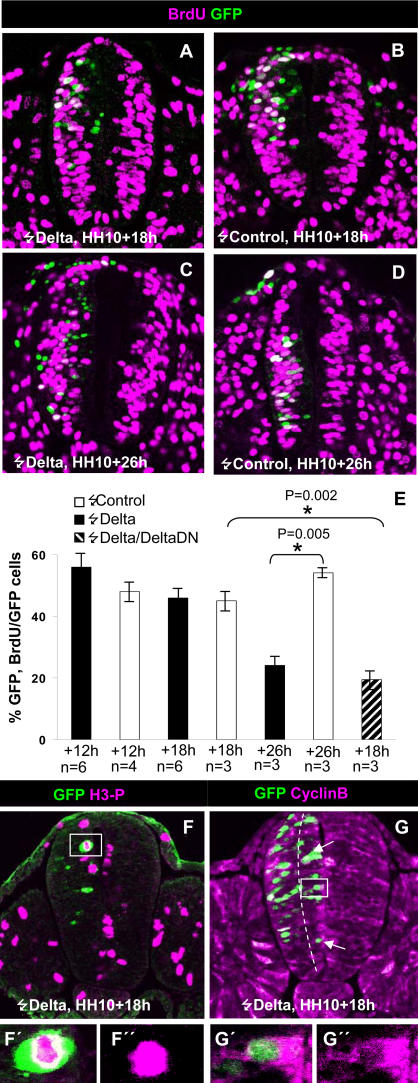
Effects of *Delta1* in proliferation of spinal cord NP progenitor cells. *A–D. HH10 embryos were transfected with either pCIG-Delta-1,* pCIG*-Delta*-1/pCIG-*Delta^DN^* or empty pCIG vector (controls) and allowed to develop for the indicated time. One hour before dissection, embryos were pulsed for 1 h with BrdU. Images show 5 µm optical sections from transversal vibratome sections taken at the level of somites 12th–16th counted from rostral. E. Statistical analysis of GFP/BrdU double labelled cells in the transfected embryos, as indicated. F,G. Double immunolabeling with PH3/GFP and Cyclin B/GFP in embryos transfected with pCIG-*Delta-1* at 18 h postransfection. F′,G′. High magnification of the framed areas in F and G showing double labelled cells. Notice the Cyclin B is mainly in the cytoplasm (G′) of this cell and also according to its apico-basal position is in G2 phase (G).

It has been proposed that the upregulation of *deltaA* in proliferating neural progenitor cells of the zebrafish neural tube induced cell cycle exit and differentiation [42]. It has been also found that the expression of the intracellular domain of DELTA-1 resulted in a non proliferating senescent-like cell phenotype [43]. This made us wonder whether the delayed effect of *Delta-1* could be due to a long delay between cell cycle exit and neuronal differentiation. In order to test this possibility, the caudal spinal cord of HH10 chicken embryos was transfected with *Delta-1* and BrdU incorporation was analyzed at 12, 18 and 26 h post-transfection. The percentage of *Delta-1* transfected cells that incorporated BrdU was very similar to that of control transfected embryos at 12 h (56±5% vs. 48±4%)(not shown) and 18 h post-transfection (46±6% vs. 45±2%, Fig. 6A,B,E). Moreover, the proportion of *Delta-1* transfected cells coexpressing the mitotic marker PH3 (12±3%, Fig. 6F) and the G2 phase marker cyclin B (19±5%, Fig. 6G) 18 h after transfection were similar to those of NP cells endogenously expressing *Delta-1* (Table 1). Therefore, in contrast to the immediate cell cycle arrest caused by *Delta^DN^* (Fig. 4), *Delta-1* transfected PNTZ NP cells do not stop cell cycling. Interestingly, the percentage of *Delta-1* transfected cells in the PNTZ that incorporated BrdU diminished substantially after 26 h (23±3% vs. 53±1%, Fig. 6C,D,E). Remarkably, the approximate 50% decrease in the number of cells that incorporate BrdU between 18 and 26 h post-transfection (Fig. 6E) coincides with the increase of TUJ1 labelled cells for the same period (Fig. 5L). These results can be interpreted as if *Delta-1* expressing NP cells of the PNTZ generate neurons by undergoing neurogenic cell cycles, those that give raise to a new progenitor and a neuron. This also implies that in order to generate neurons, *Delta-1* transfected NP cells of the PNTZ need to continue cycling. The fact that the inhibition of NOTCH signalling induced a cell cycle arrest of PNTZ NP cells (Fig. 4C,H) suggest that NOTCH signalling is required for *Delta-1* expressing NP to continue cycling. To assess this idea, we co-transfected the PNTZ with Delta-1 together with *Delta^DN^.* As predicted, the cotransfection resulted in a proliferation arrest (18,8±4,3%, vs. 45±2% in control embryos; Fig. 6E) and a strong decrease in the production of neurons (16,4±3%; Fig. 5I) 26 h post-transfection as compared with the effect of *Delta-1* alone (Fig. 5L).

Together, these experiments show that *Delta-1* expression in PNTZ NP cells is necessary and sufficient to induce neuronal generation. In addition, these results suggest that neuronal production takes place through neurogenic cell cycles rather than by inducing neuronal differentiation of the NP cells.

### DELTA-NOTCH signalling regulates the expression of *Tis21*

**Figure 7:**
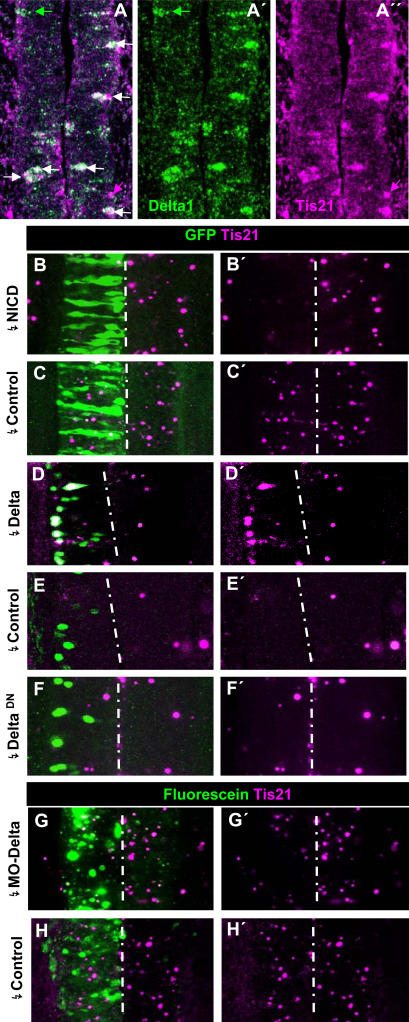
Relation of DELTA-1/NOTCH signalling and *Tis21* expression in the prospective spinal cord. A–A″. Confocal projection (25 µm) of double ISH for *Delta-1* and *Tis21* in the transition zone of a HH10 embryo showing several double labelled cells (white arrows) and cells expressing either *Tis21* (magenta arrows) or *Delta-1* (green arrows). B,C. Confocal projection (50 µm) over the PNTZ of embryos electroporated with NICD and control vector at HH10 and allowed to develop until HH12/13. D–F. Confocal projection (25 µm) around the 6 most caudal somites of embryos electroporated with pCIG-*Delta-1*, pCIG (controls) and pCIG-*Delta^DN^* at HH10 and allowed to develop until HH12/13. G,H. Confocal projection (50 µm) around the PNTZ of embryos electroporated with MO2-c*Delta1* anti-sense and control morpholinos. Notice the decrease in the number of cells expressing *Tis21* and their expression level in the side transfected with MO2-c*Delta1* as compared to the contralateral one.

In order to assess a possible switch to neurogenic NP cells in response to *Delta-1*, we examined the expression of *BTG2/PC3/Tis21*, a molecular marker of neurogenically dividing NP cells [44], [45], which we have previously found to be expressed in the developing spinal cord preceding the appearance of neurons [46]. Double ISH of chick embryos indicated that *Tis21* and *Delta-1* are indeed co-expressed in caudal spinal cord NP cells (Fig. 7A). We found that among the labelled cells located within the PNTZ of 3 embryos, 71% co-expressed both genes, while 23% expressed only *Delta-1* and 6% expressed *Tis21* alone. Accordingly, we next assessed whether *Tis21* expression could be regulated by DELTA-1-NOTCH signalling. As shown in Fig. 7BC, *Tis21* expression was extensively suppressed by electroporation with NICD (6/6 embryos). Conversely, the electroporation with pCIG-*Delta-1* of the caudal spinal cord of HH10 embryos, around prospective somites 16–20 that practically lack *Tis21* expression [46] induced ectopic *Tis21* expression beginning at 11 h (2/3 embryos, 12% of transfected cells, not shown) and highly increasing at 15 h post-transfection (10/11 embryos, 51% of transfected cells; Fig. 7D,E). Conversely, the electroporation of the PNTZ with *Delta-1* antisense morpholinos resulted in clear inhibition of *Tis21* expression (7/8 embryos, Fig. 7G,H). We also found that the suppression of NOTCH signalling by *Delta^DN^* was not capable of inducing consistent expression of *Tis21* after the same time (8/9 embryos, Fig. 7F). Altogether, these experiments demonstrate that DELTA1-NOTCH signalling in the PNTZ regulates the expression of *Tis21.*

**Figure 8:**
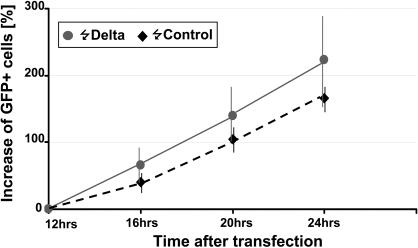
Analysis of the effect of *Delta-1* expression on cell cycle duration of spinal cord progenitor cells. HH10 chick embryos (n = 7) were transfected by electroporation with either pCIG-*Delta-1* or empty pCIG vectors and incubated for 12 hours. Then, GFP-expressing cells were counted every 4 hours in the spinal cord region around the 12th–13th somites counted from the rostral side. Results are presented as the increment of the percentage of labeled cells related to the number at 12 h posttransfection. Error bars correspond to standard deviation. Notice that data points of *Delta1*-expressing and control cells mostly fall within the respective error bars. Therefore, there is no statistically significant difference between the two curves.

It is known that in the mammalian CNS neuroepithelium there is an increase in the length of the cell cycle concomitant with the switch from proliferative to neurogenic divisions [47]. Since former studies have estimated that the duration of the cell cycle in the developing chick spinal cord is in the range of 6–8 h [1], [48], the generation of neurons 16 h after *Delta-1* expression could be explained if *Delta-1* were to drive the PNTZ NP cell into a long neurogenic cycle. However, we found that the expression of *Delta-1* in NP cells of the prospective spinal cord does not increase the duration of the cell cycle as measured by *in vivo* monitorisation of GFP transfected cells (Fig. 8).

## Discussion

### DELTA/NOTCH signalling at the transition from proliferation to neurogenesis of neural progenitor cells

The regulation of the balance between cell proliferation and differentiation is essential for the correct growth, shaping, and evolutionary diversification of the nervous system [2]–[5], [49], [50]. Thus, the switch from proliferative to neurogenic divisions of NP cells appears as a key regulatory point. Despite its biological relevance, the molecular processes that govern this switch have remained elusive. We have taken advantage of the sequential separation of the cellular processes of proliferation and neurogenesis in the prospective spinal cord of chick embryo to study the mechanisms that regulates this transition.

The chick spinal cord is generated in a rostrocaudal sequence as the body axis extends during embryonic development. This growth relies on the generation of NP cells from a region known as the caudal neural plate or stem zone, which moves caudally by regressing alongside the primitive streak. NP cells are generated in the stem zone and are left behind to form the spinal cord [22]. This process is promoted by a caudal FGF signalling gradient while an opposing rostral gradient of retinoic acid is required for neuronal differentiation [reviewed in 26]. It has been shown that FGF dependent NOTCH signalling regulates the growth of the caudal stem zone [21]. In this region, all cells express high levels of *Delta-1* and *Notch.* As a consequence, there is mutual activation of NOTCH signalling which maintains proliferation of this pool of uncommitted progenitors. In contrast, in the rostral prospective spinal cord, individual cells that express *Delta-1* differentiate into neurons and induce NOTCH signalling in neighbouring cells, which are thereby prevented from differentiating and continue to proliferate [20]. Diverse experimental approaches in chicken [37], [38], [51], [52], zebra fish [42], [53], [54], Xenopus [55], [56], and mice [57]–[61] have provided evidence to support this role of DELTA-NOTCH signalling in neuronal differentiation.

It has been proposed that as new neuroepithelium is generated immediately rostral to the caudal stem zone, there is a “transition zone” with a gradual change from mutual inhibition between all *Delta-1/Notch*-expressing cells of the caudal neural plate to lateral inhibition between single *Delta-1* expressing prospective neurons and adjacent progenitors [21].

**Figure 9:**
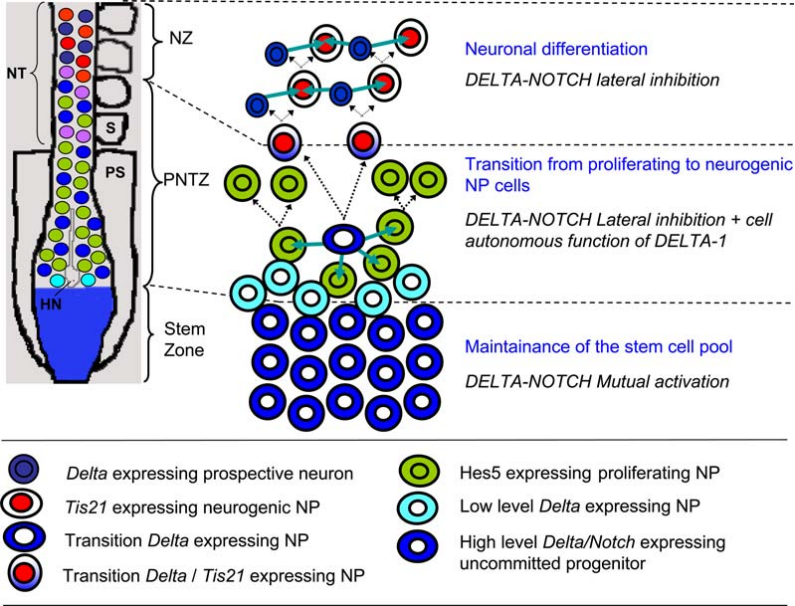
A working model for the involvement of DELTA-NOTCH signalling in the transition from proliferation to neurogenesis in the developing chick spinal cord. Schematic model of the embryonic rostro-caudal gradient of neurogenesis along the prospective spinal cord from the stem zone to the neurogenic neural tube summarising the cellular processes that seem to occur during the transition from proliferation to neurogenesis and how DELTA-NOTCH signalling may be involved in these processes. As a consequence of the caudal to rostral decreasing FGF gradient, *Delta-1* expression decreases in those cells that leave the stem zone (light blue) and move into the PNTZ where they intermingle with cells that do not express *Delta-1* [21]. This can generate differences in DELTA/NOTCH signalling between adjacent cells that may initiate lateral inhibition. This results in the upregulation of *Delta-1* in single NP cells which signal (blue arrows) and activate NOTCH signalling in adjacent cells, which as a consequence express *Hes5* and are maintained in a proliferating state. Subsequently, the *Delta-1* expressing NP cell divides into two cells that express *Tis21*. These double *Delta-1*/*Tis21* labelled NP down regulate the expression of *Delta-1* as they reach the NZ and begin to divide in a neurogenic manner. One of the daughter cells upregulates *Delta-1* expression and differentiates as a neuron while the other one, which receives NOTCH signalling (blue arrows), remains as neurogenic NP. Hensen node (HN), neural tube (NT), neurogenic zone (NZ), proliferation to neurogenesis transition zone (PNTZ), presomitic territory (PS), somite (S).

We have taken advantage of the sequential separation of the cellular processes of proliferation and neurogenesis along the rostrocaudal axis to study the possible role of DELTA1-NOTCH signalling on this transition. We have found that *Delta-1* is expressed in cycling NP cells located between the *Delta-1* expressing prospective neurons of the rostral neurogenic region [20] and the *Delta-1* expressing uncommitted progenitors of the caudal neural plate [21]. Thus, the expression of *Delta-1* in these single NP cells defines an intermediate region (PNTZ) that expands more rostrally than the previously described “transition zone” [21]. Most importantly, we have found that lateral inhibition in the PNTZ occurs between cycling NP cells rather than between a NP and a prospective neuron as in the rostral NZ (see Fig. 9 for a schematic representation). It must be also emphasized that the suppression of NOTCH signalling does not elicit neuronal differentiation of PNTZ NP cells in spite of inducing a proliferation arrest. These results contrast with the overproduction of neurons observed in other vertebrate nervous tissues after *Delta^DN^* transfection [37], [38], [42], [54], including the rostral NZ of the chick spinal cord (this paper). Together, these results indicate that neuronal differentiation is not an automatic consequence of reducing NOTCH signalling in CNS progenitors but it depends on the cellular context.

The expression of *delta* genes in proliferating NP cells has been previously reported in the embryonic zebrafish neural tube [42]. This was interpreted to mean that those cells that upregulate *delta* expression, decreased NOTCH activity by the feedback loop mechanism of lateral inhibition, and were driven to exit the cell cycle and to differentiate. However this does not happen in the PNTZ because, as we have here shown, after the expression of *Delta-1* PNTZ NP continue cycling and the suppression of NOTCH signalling did not induce cell cycle exit and neuronal differentiation. Another possibility is that *Delta-1* could function to keep selected neuronal progenitors in a pre-differentiated state until rostral differentiating gradients (i.e. retinoic acid) reach them. However, two set of results rule out this possibility. First, if this would be the case, one should expect that removing *Delta-1* function will result in precocious neuronal differentiation. However, gene silencing with *Delta-1* antisense morpholinos gave the opposite result. Secondly, the transfection with *Delta-1* in the same region of older embryos in which the rostral gradient of differentiation has advanced more caudally did not result in an earlier generation of neurons. Thus, the expression of *Delta-1* in PNTZ NP cells reveals a novel function.

We have here presented compelling results showing that DELTA–NOTCH signalling in the PNTZ regulates the transition from proliferation to neurogenesis in NP cells and that this signalling is different from that of classical lateral inhibition as happens at the rostral NZ of the spinal cord. These major conclusions are based on the following evidences: I, Activation of NOTCH signalling in the PNTZ, presumably through *Hes5*, inhibits neuronal generation and is required to maintain proliferation of NP cells. II, However, in contrast to what happens in the NZ, the suppression of NOTCH signalling in the PNTZ is not sufficient to elicit neuronal differentiation. III, The expression of *Delta-1* in cycling NP cells of the PNTZ is necessary and sufficient to generate neurons. Nevertheless, instead of driving these NP to cell cycle exit and neuronal differentiation as it happens in the NZ, after the expression of *Delta-1* PNTZ NP continue cycling. IV. After cycling, approximately half of the *Delta*-expressing cells incorporate BrdU while the other half become neurons. This fits with the switch to neuron-generating divisions, those yielding one NP cell and one neuron. V, This idea is further supported by the fact that *Delta-1* expression in cycling NP cells of the PNTZ is necessary and sufficient to induce the expression of *Tis21*, a molecular marker that identifies in the mouse CNS those NP cells that have switched from proliferative to neurogenic divisions. [44], [62]. This suggest that the transition from proliferation to neurogenesis is regulated in the PNTZ by turning proliferating progenitors (i.e. self-renewing progenitors) into neurogenic progenitors.

A common feature of lateral inhibition by NOTCH signalling throughout the animal kingdom is that mediates the binary decision of adjacent cells between two alternative fates, which depend on the developmental context [9], [17]. Accordingly, one would expect that DELTA/NOTCH signalling in different organisms under similar developmental contexts should result in equivalent fate decisions. The expression of *Delta-1* that we have found in cycling NP cells of preneurogenic regions of the mouse CNS (Figure S1) supports this idea. The increase in the ratio of symmetric/asymmetric divisions of progenitor cells found in *Numb* and *Numblike* mouse mutants 60,63) and the regulation of asymmetric divisions by *Mash1* in certain spinal cord lineages [64] also fit with this hypothesis. Interestingly, NOTCH signalling [65], [66] and *Mash1* [67] seem to promote the neuronal commitment of pluripotent stem cells.

In the embryonic CNS of *Drosophila*, neuronal progenitors (neuroblasts) arise from a neuroectoderm in which all cells initially express *Delta* and *Notch*, and have the potential to become neuroblasts or epidermoblasts. Proneural genes are expressed in clusters of cells and predispose them to a neural fate. Within each cluster *Delta* is only upregulated in a single cell that becomes a neuroblast and, through NOTCH-signalling, inhibits the neighbouring cells that remain as epidermoblasts [reviewed in 7], [8]. Cells in the neuroectoderm divide symmetrically but after delamination, neuroblasts undergo repeated rounds of asymmetric divisions. Therefore, one may interpret that after DELTA/NOTCH lateral inhibition, the neuroblast changes from proliferative to neurogenic divisions. Thus, the regulation of this switch might be an evolutionary conserved function of DELTA-NOTCH signalling. Nevertheless, since we have not directly studied the pattern of division of the PNTZ NP cells, it is unclear whether DELTA–NOTCH signalling changes PNTZ progenitors to intrinsic asymmetrically dividing NP cells or it makes the daughter cells competent to respond to other signals that determine their different fates (i.e. DELTA-NOTCH lateral inhibition at the NZ).

It must be stressed that the switch to neurogenic NP cells does not happen immediately after *Delta-1* expression since the expression of *Tis21* was induced 8 h after *Delta-1* expression and the generation of neurons after 16 h. The duration of the cell cycle in the prospective spinal cord has been previously determined to be in the range of 6–8 h by double labelling experiments with BrdU and [3H]-Tymidine [1], [48]. This fits very well with our *in ovo* measurements of the time required to duplicate the number of GFP-expressing cells in the PNTZ (Fig. 8) and contrast to the results of a recent report that found cell cycles to last 12–24 h based on time lapse determinations in the spinal cord [68]. The fact that this last study was carried out in cultured transversal slices of spinal cord that are deprived of the rostro-caudal signalling factors, which are known to stimulate proliferation, might explain the timing discrepancies.

Since *Delta-1* does not seem to increase the length of the cell cycle (Fig. 8), 16 h allow two cells cycles to pass from *Delta-1* expression to neuronal birth. Thus, the delay in the production of neurons could be explained if *Delta-1* were to drive NP cells of the PNTZ into neurogenic cell cycles after an intermediate cycle. This intermediate cell cycle might be required to rearrange the cell machinery of proliferating NP cells through the activity of possible mediators of neurogenic competence induced in response to DELTA1-NOTCH signalling.

Thus, our data fits with a model (Fig. 9) in which the single *Delta-1* expressing NP cell divides into two new NP cells, which in turn divide in a neurogenic manner. Concomitantly, DELTA-1 activates by lateral inhibition NOTCH signalling (as indicated by *Hes5* expression) in the neighbouring NP cells of the PNTZ, which remain proliferating (self-replicating). *Tis21* transcription has been reported to begin in G1 and stops at the beginning of S-phase of mouse NP [44]. Our former results in the chick spinal cord are in agreement with this [46]. Thus, our observation that *Tis21* is strongly induced 8 h (the approximate duration of one cell cycle) after *Delta-1* expression, suggest that *Tis21* expression begins after the *Delta-1* NP divide. Thus, the resulting daughter NP cells will coexpress *Delta-1* and *Tis21.* This *fits* with the high proportion of *Delta-1*/*Tis21* double labelled cells in the PNTZ. Nevertheless, *Delta-1* expression probably needs to be down regulated in these NP before they reach the NZ since in this region high levels of *Delta-1* are detected in prospective neurons rather than in neurogenic progenitors [20].

Identifying the diverse cell populations that are involved in the sequential steps of the neurogenesis process is crucial to understanding the underlying molecular mechanisms. This goal has remained elusive by the intermingling of the diverse cell types in the neuroepithelia and the shortage of specific markers. Our results can provide some molecular markers that might help to discriminate among different progenitor pools in the developing CNS. For instance, it has been shown that some HES proteins are required for maintenance of the undifferentiated state of NP cells [59], [65]. Thus, Hes5 expression could label self-replicating NP cells in this context. Similarly, *Hes5* expression seems to identify self-replicating multipotent progenitors in the embryonic mouse nervous system [69]. Additionally, we propose that the expression of *Delta-1* in single progenitors of preneurogenic neuroepithelia may identify NP cells that are switching from a proliferative to neurogenic state while the co-expression of *Delta-1* and *Tis21* may label those NP that are ready to begin to generate neurons.

It must be highlighted that the way in which DELTA1-NOTCH signalling regulates the switch from proliferative to neurogenic NPs does not seem to occur through a standard lateral inhibition as demonstrated by the fact that the suppression of NOTCH signalling by *Delta^DN^* induces neither the expression of *Tis21* nor the production of neurons. Thus, in addition to its role as NOTCH ligand in maintaining the self-replicating state of the adjacent NP cells, our results indicate that *Delta-1* may have a cell autonomous contribution to the switch to neurogenic NP cells as indicated by the cell autonomous induction of *Tis21*. Nevertheless, this process can not be regulated exclusively by cell autonomous effects of *Delta-1* since inhibition of NOTCH signalling blocks the transition to neurogenic NP cells as indicated by the reversion of the neurogenic effect of *Delta-1* by co-transfection with *Delta-DN*. Thus, NOTCH signalling is also required for this transition. Together, our results indicate that cell autonomous effects of *Delta-1* act concomitantly with NOTCH signalling to regulate this transition.

The way how *Delta-1* may act cell-autonomously in this context remains to be studied. Nevertheless, it is known that high level expression of NOTCH ligands can produce cell-autonomous inhibition of NOTCH signalling [36], [70], [71]. Interestingly, it has been found that *Delta-like-3* promotes primary neurogenesis in *Xenopus laevis* by suppressing NOTCH signalling in a cell autonomous manner [72]. However, the effects of *Delta-1* in the PNTZ can not be explained by a cell autonomous reduction of NOTCH signalling since we have found that the inhibition of NOTCH signalling in a cell-autonomous manner by *Delta^DN^* does not yield neurons.

NOTCH ligands have been for long time considered unable to transmit signals in the cells where they are expressed. However, evidences supporting a signalling role of these ligands have recently been accumulating. For instance, it has been shown that ADAM protease and γ-secretase can release an intracellular domain of *Delta,* which can be localized in the nucleus [73]–[75]. Furthermore, the over-expression of this intracellular domain in cultured neural stem cells induced neurons [75]. Thus, these observations strongly suggest the involvement of DELTA-1 mediated signalling on neurogenesis and help to build a hypothesis for its possible implication on the transition from proliferative to neurogenic NP cells. Although, we have not approached here the molecular mechanisms underlying this signalling, we have identified *Tis21* as a possible downstream mediator. The possibility that DELTA-1/NOTCH signalling triggers the switch from proliferative to neurogenic NP through activation of *Tis21* is an attractive working hypothesis that is supported by the precocious increase in the production of neurons in transgenic mice overexpressing *Tis21* [76]. .

In addition to unravelling this novel function of DELTA–NOTCH signalling in the PNTZ, our data suggest that the balance between neural proliferation and differentiation in the developing spinal cord is regulated by the sequential use of NOTCH signalling in three consecutive cellular contexts: proliferation of uncommitted progenitors, switch from proliferative to neurogenic NP cells, and neuronal differentiation (Fig. 9). It will be very interesting to uncover the molecular mechanisms that regulate DELTA/NOTCH signalling in these three sequential cellular domains and how they are coordinated within the overall process of neurogenesis.

## Materials and Methods

### Embryos

Normal fertilized chicken eggs (Gallus domesticus) were incubated at 38°C until they had reached the desired stage [77]. In some experiments, mouse embryos of the ICR strain were used.

### In ovo electroporation

Plasmid DNA (1–2 µg/µl) was injected into the neural tube of HH 10-12 chicken embryos. Two platinum electrodes were placed in parallel on either side of the neural tube, at a distance of 5mm from one another, and the embryos were pulsed 5 times (30–40 V/50 ms) using an Intrasept TSS10 pulse stimulator (Intracell). The DNA concentration and pulse voltage were adjusted depending on the desired transfection efficiency. After electroporation, the embryos were incubated at 38°C. Transfection efficiency was tested by in vivo observation of GFP or Fluorescein fluorescence under a microscope 4–12 h post-transfection. After further incubation, the embryos were either BrdU labelled or immediately fixed and processed for immunocytochemistry or FISH as described below.

We transfected a full coding sequence *cDelta-1* cDNA and a truncated version (*Delta^DN^*) lacking all but 13 of the amino acids in the intracellular region [37], [55] cloned into pCIG, a bicistronic vector that coexpresses nuclear GFP [78]. The intracellular domain of NOTCH (NICD) was cloned into the pEVRF vector [79]. In order to control the transfection efficiency, the GFP containing EGFPN1 vector (Clontech) was co-transfected together with pEVFR-NICD.

### Posttranscriptional gene silencing of *Delta-1*

We used two 25-mers fluorescein-labelled morpholino anti-sense oligos (Mo1-cDelta1, GCGTCAGCAGGAAGCGGCCTCCCAT and Mo2-cDelta1, GCGTTCCTGCCCCTGTGTCTTCGTG). These are complementary to two non-overlaping segments of sequence located around the translation start site (NCBI, Gallus gallus genome, Chromosome 3, GeneID: 395820, NW_001471668, positions 1153691–1153811). Microinjection and focal electroporation of the morpholinos in HH10 chick embryos were carried out according to experimental conditions previously described in detail by others [80] Both anti-sense morpholinos interfered with *Delta-1* function although Mo2-cDelta1 was more efficient (not shown). As a control morpholino we used a 25-mer fluorescein-labelled morpholino having a sequence (CCT CTTACCTCAGTTACAATTTATA) of the mutated β-globin of human thalassemia patients that is the standard control morpholino used in chicken [80]. All morpholinos were purchashed from GeneTools, LLC. After transfection, and fixation of embryos after appropriate incubation times, transfected cells were detected with anti-fluorescein-POD antibody (ROCHE) and TSA Plus Fluorescence System (Perkin Elmer).

### In situ Hybridisation and Immunocytochemistry

Chicken embryos were fixed with 4% paraformaldehyde for 3 hrs at RT. Whole mount *in situ* hybridization (ISH) with RNA probes for *cDelta11*, *cHairy1* and *cHairy2* was performed essentially as previously described [20], [81]. The *cHes5.1* mRNA probe was prepared from a cDNA clone (ChEST295o19, ARK-Genomics) that contains the *Hes5.1* sequence between 778–2179 bp (Accession number XM_417554). For the *Tis21* RNA probe, a 709 bp fragment of an EST clone (ptr1.pk001.n8, University of Delaware Chick EST project) corresponding to bp 1–709 of the predicted Gallus gallus *Tis21* sequence (Accession number XM_418053) was subcloned into pBluescript SK+.

Double fluorescent in situ hybridization (FISH) was carried with DIG- and fluorescein- labelled RNA, which were detected with the TSA Plus Fluorescence System (Perkin Elmer) following standard protocols. ISH was performed on whole mount embryos and 50 µm vibratome sections were then obtained to facilitate the immunocytochemical analysis. When combined with immunocytochemical detection of proteins, ISH was performed at lower temperature (52°C), lower salt concentration (SSC 1,3×), and pH = 5.0) or alternatively, using DNA probes that were generated by PCR using two templates of the *cDelta1* cDNA (678–914 bp and 1085–1425 bp: Access N° U26590), and of *Tis21* as previously described [46].

Conditions for the use of antisera against GFP (Invitrogen), phosphorylated-histone H3 (PH3) and cyclin D (Upstate Biotechnology), cyclin B1 (clone V152, Abcam), p27KIP1 (clone 57,BD Transduction Laboratories), neuronal class III β-tubulin (TUJ1, Covance) were optimized. Cy2, Cy3, and Cy5-conjugated secondary antibodies were used as recommended by the supplier (Jackson Immunochemicals Ltd). Images were acquired on a Leica TCS-SL spectral confocal microscope.

### BrdU labelling and cell cycle analysis

Proliferating cells were detected in chick embryos by in ovo incorporation of BrdU. Thus, 50 µl of a 5 mg/ml solution of BrdU in PBS was applied to the top of the embryo after opening a window in the eggshell. After incubation (20 min–4 h), the embryos were fixed as described above. Immunodetection of BrdU labelled cells was carried out on 40 µm vibratome sections with anti-BrdU (Becton Dickinson) and detected with Cy3-conjugated secondary antibody. When ISH was combined with BrdU labelling, denaturation of DNA by treatment with 2 N HCl for 30 min was carried out after, DIG immunolabelling and before the subsequent BrdU immunodetection.

The analysis of the expression of *Delta-1* at the different stages of the cell cycle was based on the use of the appropriate markers and the differential apical-basal position of the nuclei. Thus, replication of DNA during S-phase takes place in the basal half of the neuroepithelium; during G2, the nuclei move towards the ventricular surface where mitosis takes place; during G1 nuclei move towards the basal region [29]. Mitotic cells were labelled with anti-phosphohistone-H3. Anti-cyclin D was used to detect cells in G1 phase. To assess the expression of *Delta1* during the S-phase, embryos were exposed to a pulse of BrdU for a very short period of time (20 min) in order to avoid labelled cells moving to G2 by the end of the pulse. Accordingly, only cells in the basal half of the neuroepithelium were labelled under these conditions. Anti-cyclin B was used to label cells in G2 phase. Since Cyclin B is expressed in the cytoplasm during the S and G2-phases and it translocates into the nucleus during mitosis [82], statistical counts of double labelled cells focused only on cells containing cytoplamic Cyclin B and being located within the apical third of the neuroepithelium where practically no BrdU-labelled cells were found after a short pulse. Cell counting was carried out over single optical confocal sections. The mean proportion of cells co-expressing specific genes was obtained for each embryo and the error calculated as the standard deviation. The statistical significance (P value) between experimental and control samples was determined using the Student's t-test.

In vivo analysis of cell cycle duration was carried on HH10 chick embryos electroporated with either pCIG-*Delta-1* or pCIG. The time required for GFP expressing cells to double their number was determined by counting labelled cells in the neural tube region between somites 12–13th with a Leica MZFLIII stereo microscope using a 2× magnification objective. To make sure that our estimation of the increase in the number of GFP labelled cells with time was due to cell division and not to upregulation of GFP expression, the determination was started at 12 h post-transfection (i.e. 5 h from beginning of GFP expression). Also, in order to avoid dilution of GFP signal by cell division, cell counting was not carried after 24 h post-transfection.

## Supporting Information

Figure S1 Coexpression of Delta1 and cyclin D in early mouse neuroepithelium.
(0.13 MB PDF)

Figure S2 Expression pattern of Hes genes in the developing caudal spinal cord of chick embryos.
(0.07 MB PDF)

Figure S3 Control electroporation experiments.
(0.52 MB PDF)
