# Supplementary material for: A Novel Function of DELTA-NOTCH Signalling Mediates the Transition from Proliferation to Neurogenesis in Neural Progenitor Cells
Source: PLoS One. 2007 Nov 14;2(11):e1169. doi: 10.1371/journal.pone.0001169 (PMC2064965; doi:10.1371/journal.pone.0001169)
Supplement: Figure S1 — Coexpression of Delta1 and cyclin D in early mouse neuroepithelium. (0.13 MB PDF) [file pone.0001169.s001.pdf]

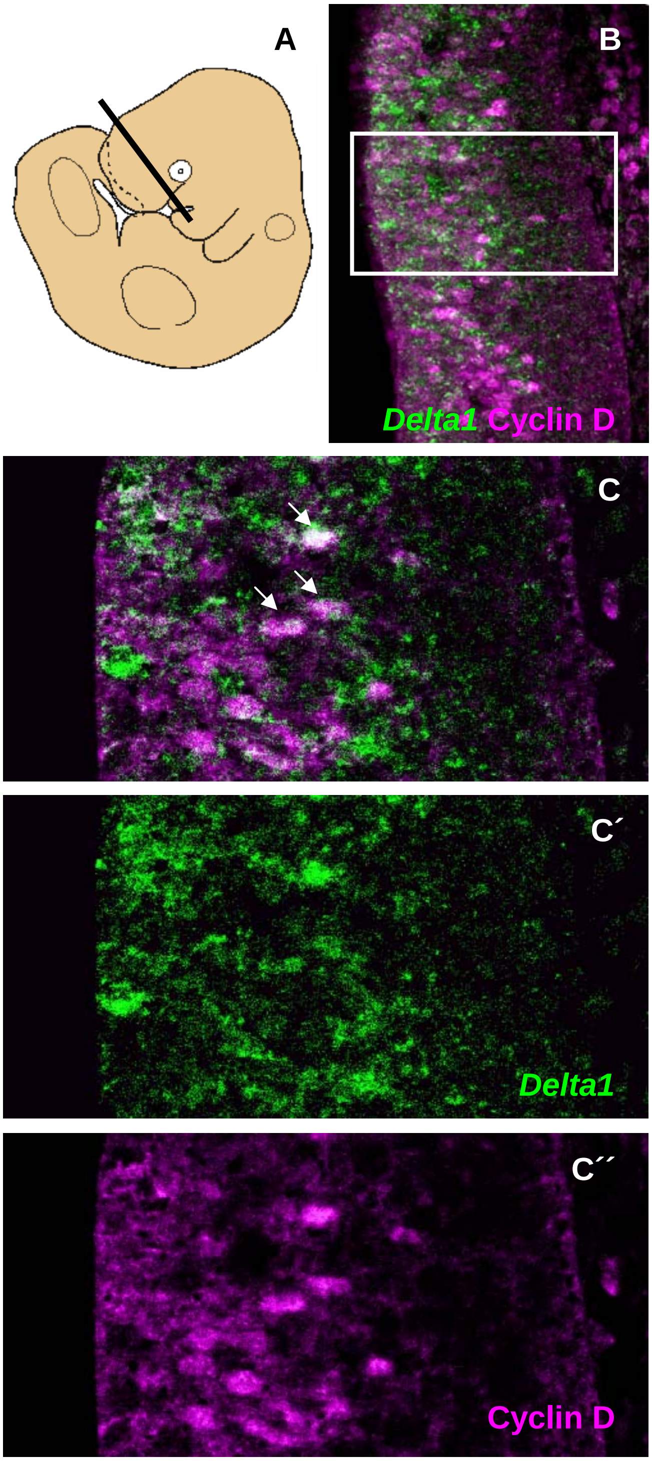

Figure S1. Coexpression of Delta1 and cyclin D in early mouse neuroepithelium. Double immunolabelling of *Delta-1* mRNA and Cyclin D in coronal forebrain sections of E10.5 mouse embryos. A. Schematic representation of the embryo indicating the approximate position where the section was obtained. B, Confocal projection (85µm). C-C´´ Confocal optical section (5µm) of the boxed area in B. Double labelled cells are indicated by arrows.
