# Supplementary material for: A Novel Function of DELTA-NOTCH Signalling Mediates the Transition from Proliferation to Neurogenesis in Neural Progenitor Cells
Source: PLoS One. 2007 Nov 14;2(11):e1169. doi: 10.1371/journal.pone.0001169 (PMC2064965; doi:10.1371/journal.pone.0001169)
Supplement: Figure S2 — Expression pattern of Hes genes in the developing caudal spinal cord of chick embryos. (0.07 MB PDF) [file pone.0001169.s002.pdf]

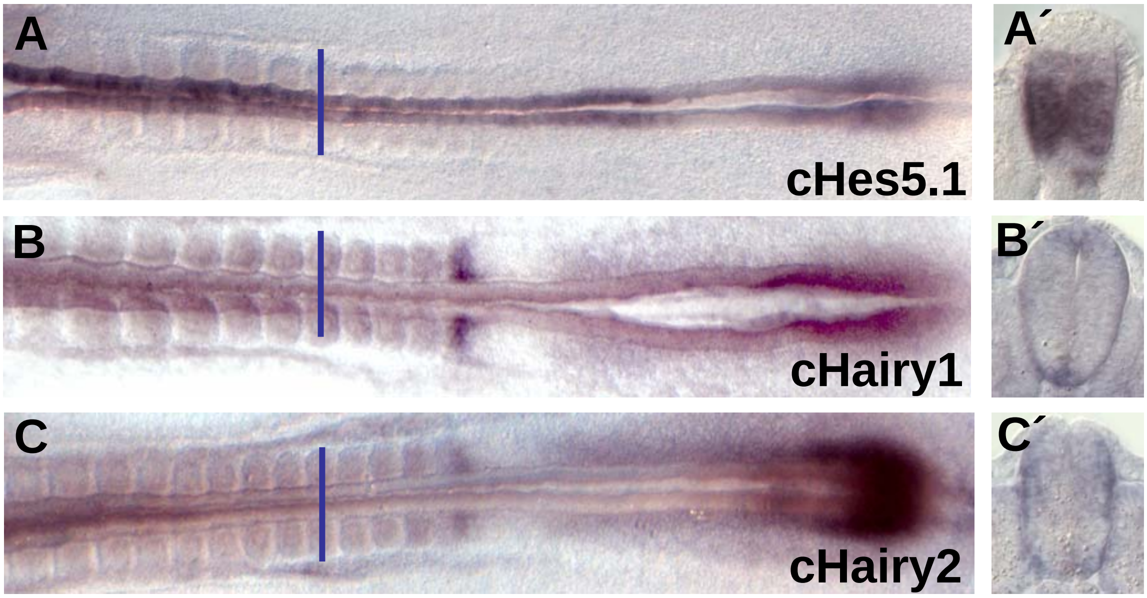

Figure S2. Expression pattern of *Hes* genes in the developing caudal spinal cord of chick embryos. A-C. ISH of *Hes5.1, cHairy1 and cHairy2* carried out in a HH10/11 chick embryos showing expression in the caudal neural plate, most caudal somites, and prospective spinal cord. A'-C'. Transversal sections taken from the respective whole mount ISHs (A-C) at the approximate positions indicated by blue lines.
