# Supplementary material for: A Novel Function of DELTA-NOTCH Signalling Mediates the Transition from Proliferation to Neurogenesis in Neural Progenitor Cells
Source: PLoS One. 2007 Nov 14;2(11):e1169. doi: 10.1371/journal.pone.0001169 (PMC2064965; doi:10.1371/journal.pone.0001169)
Supplement: Figure S3 — Control electroporation experiments. (0.52 MB PDF) [file pone.0001169.s003.pdf]

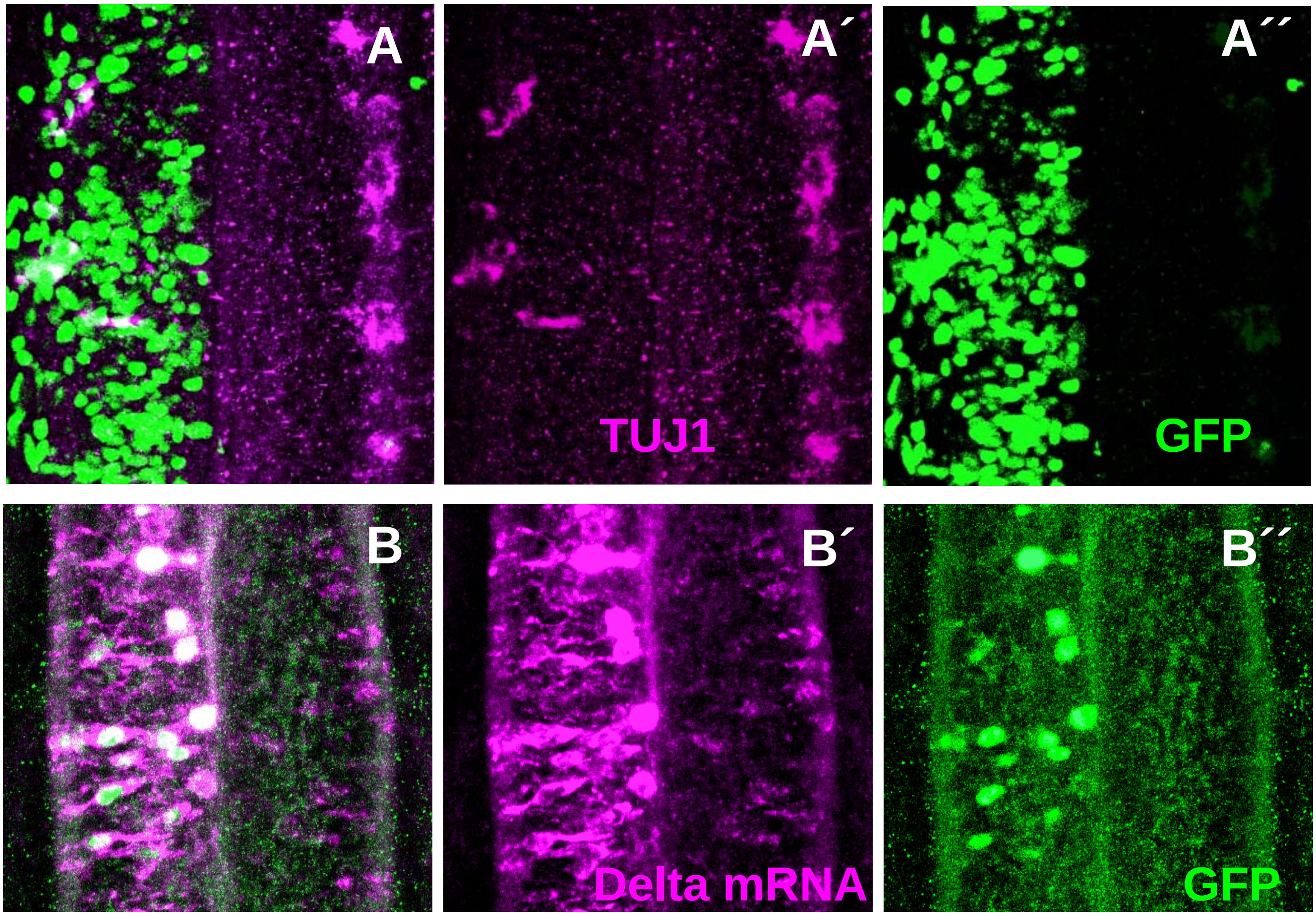

Figure S3. Control electroporation experiments. A-A´´. Double GFP/TUJ1 immunolabelling carried out on embryos massively transfected with Delta1-pCIG at 26h postransfection. Images show confocal Z-axis projections over 50µm. Notice the decrease in the number of TUJ1-labeled cells in the transfected side of the neural tube. B-B''. GFP immunolabelling combined with *Delta1* ISH carried out on embryos transfected with Delta1-pCIG at 8h postransfection. Images show confocal Z-axis projections over 50µm. Notice that most GFP expressing cells co-express *Delta1* mRNA.
